# Public health engagement: detection of suspicious skin lesions, screening and referral behaviour of UK based chiropractors

**DOI:** 10.1186/s12998-014-0047-2

**Published:** 2015-01-23

**Authors:** Sara Glithro, David Newell, Lorna Burrows, Adrian Hunnisett, Christina Cunliffe

**Affiliations:** McTimoney College of Chiropractic, Abingdon, UK; Anglo European College of Chiropractic (AECC) and Bournemouth University, Dorset, UK; Salisbury NHS Foundation Trust, Salisbury, UK

**Keywords:** Skin lesion, Skin cancer, Screening, Referral behaviour, Prevention, Detection, Dermatology, Chiropractor, Manual therapist

## Abstract

**Background:**

UK morbidity and mortality rates from skin cancer are increasing despite existing preventative strategies involving education and early detection. Manual therapists are ideally placed to support these goals as they see greater quantities of exposed patient skin more often than most other healthcare professionals. The purpose of this study therefore was to ascertain the ability of manual therapists to detect, screen and refer suspicious skin lesions.

**Method:**

A web-based questionnaire and quiz was used in a sample of UK chiropractic student clinicians and registered chiropractors to gather data during 2011 concerning skin screening and referral behaviors for suspicious skin lesions.

**Results:**

A total of 120 questionnaires were included. Eighty one percent of participants agreed that screening for suspicious skin lesions was part of their clinical role, with nearly all (94%) assessing their patients for lesions during examination. Over 90% of the participants reported regularly having the opportunity for skin examination; with nearly all (98%) agreeing they would refer patients with suspicious skin lesions to a medical practitioner. A third of respondents had referred a total of 80 suspicious lesions within the last 12 months with 67% warranting further investigation.

**Conclusions:**

Nearly all respondents agreed that screening patients for suspicious skin lesions was part of their clinical role, with a significant number already referring patients with lesions.

## Background

Malignant melanoma incidence rates have increased more than fivefold since the mid-1970s and continue to rise [1]. In 2002, the UK paid over £240 million in total costs associated with skin cancer; of which 42% were direct costs to the NHS [2]. Of the various forms of skin cancer, melanoma treatment was directly responsible for 63% of this total. Early detection of pre-cancerous and cancerous lesions not only improves patient mortality rates [3-5] it also substantially reduces this economic burden by enabling faster, cheaper and less-invasive interventions as out-patients in comparison to the otherwise high costs associated with in-patient care required to treat advanced lesions. Yet, in a study of US patients, 75% reported having never been advised by their medical practitioner or any other medical professional in their medical practice, to examine their skin for growths or changes in spots or moles. Furthermore, most participants (58% and 83% respectively) reported that their medical practitioner ‘never’ or ‘rarely’ looked at the skin on their back or on the backs of their legs [6].

However, in the UK, in addition to medical practitioners, a diverse group of clinical practitioners are in a position to detect, screen and refer suspicious skin lesions [6-10]. Of these, a smaller number regularly see patients unclothed and particularly see areas of the body inaccessible to visual inspection by the patient themselves. Of those that do, manual therapists such as masseurs, osteopaths and chiropractors are ideally placed to regularly screen the skin for suspicious lesions. Chiropractic practice is concerned with the diagnosis and treatment of musculoskeletal syndromes, including low back, neck pain, headaches and peripheral joint problems [11]. Despite 42% of chiropractors regularly examining patients who were in their underwear [12], little if any research exists that has attempted to determine whether chiropractors in the field take advantage of this opportunity to perform basic skin screening. Of that research already carried out, this was restricted to a student population only and recommended that further research should test the ability of chiropractors to identify pathological moles on a broader, evaluative basis [13]. In view of this we investigated the accuracy with which chiropractic student clinicians and registered chiropractors were able to recognise as suspicious or benign, a variety of common skin lesions from images chosen by a dermatologist. More generally we also examined attitudes towards skin screening and the screening and referral patterns within this sample. For the purpose of this article the term ‘chiropractic student clinicians’ has been adopted to describe chiropractic students or interns in their final year of clinical study.

## Methods

### Dissemination

A web based, self-administered questionnaire was emailed to the chiropractic student clinicians of both participating UK colleges. These participants were in their final clinical year of study when the survey was distributed in 2011 (n = 154). The questionnaire was also emailed to a sample of 250 practising UK registered chiropractors who were randomly selected by the McTimoney Chiropractic Association from their members list. Finally, a link to the questionnaire was published in the United Chiropractic Association’s regular members’ e-newsletter. The questionnaire contained closed questions and images designed to gather descriptive information on the participants’ frequency and accuracy of detecting suspicious skin lesions.

### Inclusion/exclusion criteria

Students were those in their final clinical year of chiropractic study at a UK college or university which on completion of their course, allowed registration with the General Chiropractic Council (GCC). The chiropractors were all registered with the GCC at the time the questionnaire was issued. For inclusion within the data analysis, respondents must have confirmed whether they were either a student or a registered chiropractor. In addition, they had to provide a response to the question concerning whether or not they considered screening patients for suspicious skin lesions to be part of their role as a chiropractor. Only subjects that responded either ‘Yes’ or ‘No’ were included as failure to answer this question meant the subject had exited the questionnaire without answering any further questions. Questionnaire completion was advised and taken as consent.

### Questionnaire design

The questionnaire had four sections: 1) Participant demographics, 2) Willingness and attitudes towards examination of patients for skin lesions, 3) Skills associated with lesion detection and 4) Identification of training needs associated with lesion detection.

Likert scales were used to evaluate participants’ knowledge of risk factors involved with and identification of, suspicious skin lesions. The second part of the questionnaire established participants’ skills in identifying lesions. A dermatologist advised on and approved a set of images of commonly occurring skin lesions, including some where early detection could have substantial impact on prognosis and decrease costs to health services. In an attempt to prevent participants from searching for the images online prior to answering questions; a statement encouraging the participants to resist using any reference material was displayed at the beginning of the questionnaire; image references were only revealed to the participants on questionnaire completion and an on-demand document providing all the images, references and correct diagnoses was also provided to participants on questionnaire completion. Face validity and piloting of the questionnaire took place prior to finalising the questionnaire design. Anonymous responses were accepted for a period of four weeks, with emailed reminders sent at pre-identified points during this period.

### Sampling

The aim was to capture responses from a sample group reflective of the UK chiropractic profession and a final year pre-registration student chiropractic population. Invitations to take part were made to the four UK chiropractic associations; British Chiropractic Association (BCA), McTimoney Chiropractic Association (MCA), Scottish Chiropractic Association (SCA), United Chiropractic Association (UCA) and all three UK chiropractic colleges; Anglo-European College of Chiropractic (AECC), McTimoney College of Chiropractic (MCC) and the Welsh Institute of Chiropractic (WIOC).

The final sample included 62 UK based registered chiropractors who were members of the MCA and 58 final year students from two UK teaching institutions. Of these, 34 (58%) were from AECC and 25 (42%) were from the MCC.

### Analysis

Questionnaire data was analysed using a descriptive approach. Continuous variables where normally distributed were described using means and standard deviations, while non-parametric distributions were described with medians and percentiles. Categorical variables were described using proportions.

### Ethics

Ethical approval was granted by the McTimoney College Research Ethics Committee on the 21st of November 2010, prior to commencement of the study.

## Results

From the 404 questionnaires that were issued, a total of 139 questionnaires were returned. Nineteen failed to meet the inclusion criteria, leaving 120 included in the analysis and giving a successful response rate of 30%. Eighty (67%) participants were female and 40 (33%) were male. Sixty two (52%) were registered chiropractors and the remaining 58 (48%) were chiropractic students. In the registered chiropractor group, the mean number of years qualified was 5.8 years (SD ± 8.0) and the mean number of patients treated per month was 89 (SD ± 70.3) (Table 1).

**Table 1:** **Demographic characteristics of sample**

**Variable**		**n**	**%**	**Mean (SD)**	**Range**
Gender	Female	80	67		
Age (years)	21-30	36	30		
	31-40	27	23		
	41-50	31	26		
	51-60	16	13		
	>60	10	8		
Practicing category	Chiropractic student clinician	58	48		
	Registered Chiropractor	62	52		
Years qualified				5.8 (8)	0-35
	Not yet qualified	58	48		
	Less than 5 years	14	12		
	5-9 years	15	13		
	10-14 years	15	13		
	15-19 years	7	6		
	20-24 years	6	5		
	25-35 years	5	4		
New patients per month				12.8 (13)	0-108
	1-4	50	42		
	5-9	18	15		
	10-19	25	21		
	20-39	21	18		
	Over 40	5	4		
Repeat patients per month				76.1 (65)	13-347
	1-49	58	48		
	50-99	28	23		
	100-199	27	23		
	200-799	7	6		

**Figure 1:**
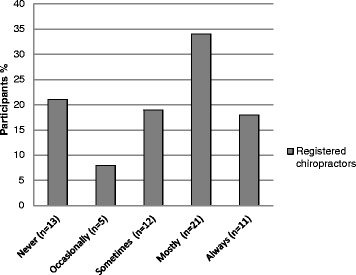
**Percentage of registered chiropractor participants who performed chiropractic examinations with patients wearing only their underwear thus having the opportunity to examine at least some of their skin.**

As the chiropractic student clinicians approach to examination of patients was likely to be dictated by their college policy on patient gowning, skin exposure analysis was evaluated from the data provided by participating registered chiropractors. Figure 1 shows the degree of patient skin exposure during the examination and treatment by the registered chiropractor. Fifty two percent (n = 32) agreed they ‘always’ or ‘mostly’ examined patients in their underwear during the examination. In another question, over half, (71%, n = 44) indicated that their patients were ‘never’ or ‘occassionally’ fully clothed during the examination, implying regular opportunities to examine at least some of their patients’ skin.

Most chiropractic student clinician and registered chiropractor participants (81%, n = 97) agreed that screening patients for skin lesions was part of their clinical role (Table 2). Of these, 94% (n = 91) indicated they screened each new patient, 53% (n = 51) screened existing patients at every visit and 73% (n = 71) at visits scheduled specifically for patient re-assessments.

**Table 2:** **Screening attitude and behaviours of participants**

**Question posed**		**Answered 'Yes'**	
		**%**	n
Do you consider screening patients for suspicious skin lesions to be part of your role as a chiropractor?		81	97
If 'Yes', is this part of your normal patient assessment for…	New patients?	94	91
	Repeat patients at every visit?	53	51
	Repeat patients during regular assessments?	73	71

**Figure 2:**
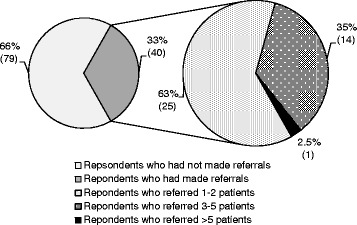
**Participants (all) referral rates of patients with suspicious skin lesions within the previous 12 months %(n).**

Registered chiropractors and final year chiropractic students already identify and refer patients with suspicious skin lesions. Within the previous 12 months, 33% (n = 40) of the participants had referred a total of 104 patients with skin lesions they felt warranted further investigation (Figure 2).

**Figure 3:**
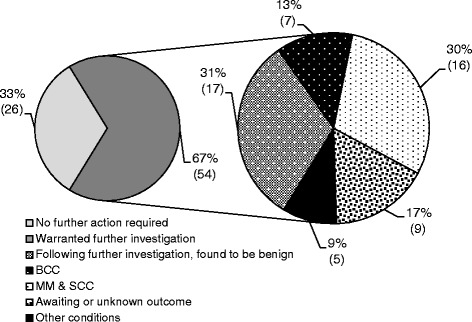
**Patient outcomes following referral %(n).**

Of the 104 referrals made, referrers declared they were aware of 80 outcomes via feedback from their patients or medical practitioners. Over two-thirds of the 80 outcomes known (67%, n = 54), warranted further secondary healthcare investigation by a medical professional. Of these 80 outcomes; 29% (n = 23), were diagnosed as Basal Cell Carcinoma, Malignant Melanoma or Squamous Cell Carcinoma. A further 17% (n = 9) of patients were still awaiting the outcome and 9% (n = 5) were diagnosed with other conditions requiring medical care. Almost a third (31%, n = 17) of those referred required no further treatment following examination by their medical practitioner (Figure 3).

The participants generally demonstrated a good understanding of the risk factors associated with developing skin cancers, although some frequently occurring features were less understood (Table 3). Few respondents identified having blue/green eyes (10%, n = 12), red hair/freckles (37%, n = 36) or an unprotected balding head (45%, n = 53) as risk factors for developing skin cancer. Yet over 88% recognised an increased risk with having an outdoor occupation, repeated sunburn as a child or history of using UV tanning beds. When assessing whether a lesion should be considered suspicious, over 90% agreed or strongly agreed the importance of a change in the appearance or behavior of a lesion. Furthermore, over 70% agreed or strongly agreed that the recent appearance, an uneven colour or lesion asymmetry should also be looked out for. Additionally, 55% deemed a raised surface to be important although this is not a major feature for all suspicious lesions. The majority practiced a proactive approach in the detection and referral of lesions however, less than 40% advised their patients on preventative strategies in avoiding or reducing risk.

**Table 3:** **Respondents knowledge of the risk factors associated with developing skin cancer and the advice they offer patients**

	**%**	**n**
**The risk of developing skin cancer increases with…'**		
An outdoor occupation		
Strongly agree/agree	76	89
Neutral	20	23
Disagree/strongly disagree	4	5
An indoor occupation and holidays in the sun once or twice a year (beach, skiing, outdoor etc.)		
Strongly agree/agree	71	82
Neutral	21	24
Disagree/strongly disagree	8	9
Repeated incidents of having sunburn as a child		
Strongly agree/agree	88	104
Neutral	11	13
Disagree/strongly disagree	1	1
A history of using UV tanning beds		
Strongly agree/agree	93	110
Neutral	6	7
Disagree/strongly disagree	1	1
More than 50 moles		
Strongly agree/agree	56	65
Neutral	38	45
Disagree/strongly disagree	6	7
More than 100 moles		
Strongly agree/agree	69	78
Neutral	27	31
Disagree/strongly disagree	4	4
A history of at least one pre-cancerous or cancerous skin lesion		
Strongly agree/agree	92	109
Neutral	6	7
Disagree/strongly disagree	2	2
A first degree relative with a history of at least one pre-cancerous or cancerous skin lesion		
Strongly agree/agree	69	82
Neutral	22	26
Disagree/strongly disagree	8	10
Freckles and/or red hair		
Strongly agree/agree	37	36
Neutral	51	49
Disagree/strongly disagree	12	12
Blue or green eyes		
Strongly agree/agree	10	12
Neutral	65	75
Disagree/strongly disagree	25	29
Fair skin		
Strongly agree/agree	58	69
Neutral	34	40
Disagree/strongly disagree	8	9
A history of long-term use of drugs with known side-effects of photosensitivity		
Strongly agree/agree	66	77
Neutral	32	37
Disagree/strongly disagree	3	3
Thinning or balding hair		
Strongly agree/agree	45	53
Neutral	42	50
Disagree/strongly disagree	13	15
**A skin lesion should be considered 'suspicious' if…**	**%**	**n**
It has uneven colour		
Strongly agree/agree	72	84
Neutral	23	27
Disagree/strongly disagree	4	5
It is asymmetrical		
Strongly agree/agree	73	85
Neutral	22	25
Disagree/strongly disagree	5	6
It has changed in size (growing)		
Strongly agree/agree	95	114
Neutral	2	2
Disagree/strongly disagree	3	4
It has changed in shape (irregular border)		
Strongly agree/agree	97	113
Neutral	2	2
Disagree/strongly disagree	1	1
It has changed in colour (including several different colours)		
Strongly agree/agree	93	112
Neutral	4	5
Disagree/strongly disagree	3	3
It has a crusty keratinised surface		
Strongly agree/agree	71	82
Neutral	22	26
Disagree/strongly disagree	7	8
It bleeds		
Strongly agree/agree	95	110
Neutral	3	3
Disagree/strongly disagree	3	3
It appeared recently		
Strongly agree/agree	71	82
Neutral	24	28
Disagree/strongly disagree	5	6
It has a raised surface		
Strongly agree/agree	55	66
Neutral	32	38
Disagree/strongly disagree	13	15
It itches		
Strongly agree/agree	78	91
Neutral	17	20
Disagree/strongly disagree	4	5
The patient is concerned about it		
Strongly agree/agree	73	85
Neutral	25	29
Disagree/strongly disagree	3	3
**Do you advise your patients on…**	**%**	**n**
The risks of sun exposure		
Yes	33	39
No	67	78
The risks of using UV sunbeds		
Yes	37	43
No	63	74
Using sunscreen with an SPF of at least 15		
Yes	32	36
No	68	78
Other factors that can increase photosensitivity (E.g. some antibiotics, anti-depressants, anti-malarial medications etc.)		
Yes	14	16
No	86	96
Have you ever been asked for advice on preventing skin cancer by your patients?		
Yes	7	8
No	93	108

**Figure 4:**
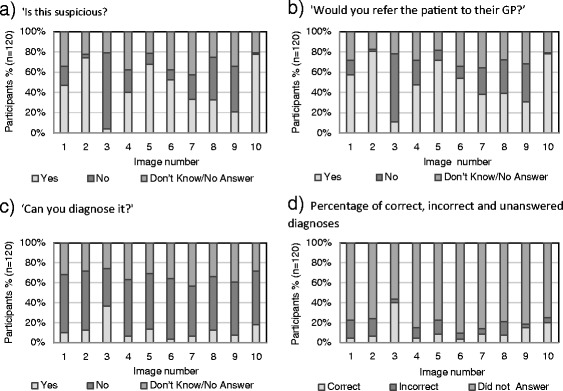
**Participants response to questions concerning images.** For **a)** to **d)**: n = 120; Images, 1: Superficial Basal Cell Carcinoma, 2: Squamous Cell Carcinoma, 3: Intradermal Naevus, 4: Actinic Keratosis, 5: Seborrhoeic Wart, 6: Bowen’s Disease, 7: Nodular Basal Cell Carcinoma, 8: Haemangioma, 9: Junctional Naevus and 10: Malignant Melanoma. NB: for the purposes of this study, images 1,2,4,6,7 and 10 were considered ‘suspicious’ by the researchers.

Figures 4a to d document the responses to questions asked about the ten images of skin lesions shown to the subjects. As is shown, with reference to identification of suspicious lesions, over 75% of participants correctly labeled examples of malignant melanoma and squamous cell carcinoma. However, less than 45% did so for superficial basal cell carcinoma and actinic keratosis. Interestingly, whilst confident to label these lesions as suspicious, less than 10% diagnosed the lesions correctly (Figure 4d). Over 60% incorrectly labeled the benign seborrhoeic wart as suspicious, however more participants correctly labeled the haemangioma and junctional naevus as not suspicious. The majority, 75% (n = 90) correctly identified the intradermal naevus as not suspicious.

Participant’s referral rates were slightly greater (<11%) than those declaring the corresponding lesion as ‘suspicious’ (Figure 4b). Whilst not wanting to over-refer, perhaps this degree of caution demonstrates respondents recognising when to request help beyond their own expertise. Participant’s confidence in their ability to diagnose the images correctly mirrored the actual number of correct diagnoses; however both were low at less than 20%.

Participants were asked whether they had completed any skin lesion identification training as part of their undergraduate course and whether they would be interested in receiving more. The majority (68%, n = 81) had received some form of undergraduate training and 75% (n = 90) of all respondents were interested in receiving further training.

## Discussion

A position paper decrying the less than main stream position of chiropractic within the wider health care system and using podiatry as a model to describe how a similar profession has navigated a course toward greater acceptance, cited podiatrists traditional dedication to public health issues as one of the major reasons why they became influential members of the healthcare community [14]. They urged the chiropractic profession to *‘…openly embrace, and become actively involved in public health initiatives’*.

In the context of these discussions this study investigated the degree to which chiropractors were engaging in an important key public health issue, namely early detection of skin cancer.

As long ago as 2003, Mahon [15] concluded that given skin cancer remaining as a major public health problem, efforts following those associated with primary prevention, such as sun screen use and judicial reductions in exposure to UV, should also include secondary prevention such as professional skin examinations. This author went on to suggest that nurses have a major role to play in these secondary preventative efforts.

A recent article by Ramcharan et al. [13] using a cross sectional design, reported that over 80% of chiropractic student clinicians thought that recognising skin cancer in their patients was important or very important in their practice, concluding that chiropractic education should emphasize the opportunity to detect and assess atypical moles as a routine part of primary prevention in clinical education.

Similarly in this study, the majority of participants agreed that screening patients was part of their clinical role and felt they already had the skills to recognise suspicious lesions and refer patients for further investigation. Within this small sample (n = 120), 40 practitioners had referred 80 patients for further investigation within the previous 12 months and at least 23 were found to have skin cancer. Extrapolating to the UK registered chiropractic population of approximately 2827, one could estimate that the chiropractic profession might identify and refer 1866 patients annually for further investigation and of these 29% (541) could have skin cancer.

This research indicates that detecting and referring patients with suspicious skin lesions is already taking place, yet patient education in primary prevention by chiropractors is lacking during these consultations. A need and willingness to undertake further training was identified which, if put in place, may improve patient education and contribute to improving outcomes in skin cancer.

Clearly the study has limitations. Firstly, generalizability is limited with the inclusion of participants from only two of the three UK chiropractic educational institutions and only one chiropractic association. This means the results may not be representative of the whole UK chiropractic population, although the one association included does comprise 19% of the UK chiropractic practitioner population. Secondly, whilst every effort was made in the research design to discourage participants, the possibility that reference material was used while completing the questionnaire cannot be overlooked.

## Conclusion

Visual screening is an important weapon in the arsenal against the rise of malignant skin lesions and is also highly cost effective [16]. Chiropractors serve to positively contribute to this public health problem and the role of the profession should both be articulated and improved in this respect.

This study has shown that not only do chiropractic student clinicians and registered chiropractors feel this screening to be an important part of their role but it also indicates that significant numbers of patients are referred on to specialist care with the potential to importantly reduce mortality and morbidity together with the substantial costs associated with these lesions.

With focused undergraduate training and further post graduate opportunities, chiropractors may feel more confident in detecting suspicious skin lesions and subsequently prove to be increasingly important advocates in the early detection of skin cancer.
